# Potential Rapid Quantification of Antioxidant Capacity of *Olea europaea* L. Leaves by Near-Infrared Spectroscopy Using Different Assays

**DOI:** 10.3390/antiox14101246

**Published:** 2025-10-17

**Authors:** Manuel Piqueras-García, Jorge F. Escobar-Talavera, María Esther Martínez-Navarro, Gonzalo L. Alonso, Rosario Sánchez-Gómez

**Affiliations:** Cátedra de Química Agrícola, Escuela Técnica Superior de Ingeniería Agronómica y de Montes y Biotecnología (ETSIAMB), Universidad de Castilla-La Mancha, Avda. de España s/n, 02071 Albacete, Spain; manuel.pgarcia@uclm.es (M.P.-G.); jorge.escobar@uclm.es (J.F.E.-T.); mesther.martinez@uclm.es (M.E.M.-N.)

**Keywords:** ABTS, DPPH, NIR calibration, olive leaf, ORAC, phenolic compounds

## Abstract

The olive tree has exceptional agricultural and economic importance in Mediterranean regions due to its fruit, which is used to produce olive oil. However, the olive oil industry generates a significant amount of waste, including leaves from *Olea europaea* L. These leaves contain a high concentration of bioactive compounds, predominantly phenolic ones, which are well known for their antioxidant properties and health benefits. Determining antioxidant capacity involves the use of different assays based on absorbance (DPPH, 2,2-diphenyl-1-picrylhydrazyl; and ABTS, 2,2′-azino-bis(3-ethylbenzothiazoline-6-sulfonic acid)) and fluorescence (ORAC, Oxygen Radical Absorbance Capacity), which require reagents and long waiting times. Therefore, having a non-destructive technique capable of providing this information would be useful. To explore this, 120 olive leaf samples were analyzed using the three antioxidant assays to quantify their total antioxidant capacity. Predictive models were successfully developed for each of the three methods, achieving coefficients of determination (R^2^) between 0.9 and 1 across calibration, validation, and prediction. Additionally, high residual predictive deviation (RPD) values were obtained, indicating that the models exhibit strong reliability and predictive performance.

## 1. Introduction

The olive tree (*Olea europaea* L.) is one of the oldest cultivated fruit trees, with a history dating back more than 5000 years in the Mediterranean region. Today, it remains a dominant species in the agricultural systems of Southern Europe, particularly in Spain, which leads global olive oil production with more than 2.6 million hectares dedicated to olive cultivation [1]. Despite the emphasis on fruit and oil, olive cultivation generates large volumes of residues, notably olive leaves. For example, in Spain, the total amount of annual leaf amounts to 1.25 million tonnes per year [2]. However, the leaves are commonly underutilized despite their promising biochemical properties [3].

Recent studies have demonstrated that olive leaves contain a diverse array of phenolic compounds, including oleuropein, hydroxytyrosol, verbascoside, luteolin-7-O-glucoside, rutin, and apigenin derivatives [4,5]. These compounds are associated with a wide range of biological effects, including antioxidant, anti-inflammatory, antihypertensive, and antimicrobial activities [6]. Among these, oleuropein is the predominant, accounting for up to 15% of the dry weight of leaves and as much as 70–75% of total phenolics [7].

Among the above effects, the antioxidant capacity is a particularly valuable metric, as it reflects the ability to scavenge reactive oxygen species (ROS). Conventional in vitro assays such as DPPH and ABTS are based on absorbance and in a free radical with a characteristic color that decreases in intensity as it is reduced, while ORAC is based on fluorescence and the ability to neutralize free radicals. All of them are widely used to quantify antioxidant activity, but these techniques are labor-intensive, require chemical reagents, and are not well-suited for high-throughput or environmentally sustainable workflows [8]. In this context, near-infrared (NIR) spectroscopy has emerged as a valuable alternative. This technique enables rapid, non-destructive analysis based on overtone and combination vibrations of molecular bonds such as O–H, N–H, and C–H functional groups, abundant in phenolic-compound-rich plant tissues [9]. The complexity and overlapping nature of NIR spectra necessitate the use of chemometric techniques, especially Partial Least Squares Regression (PLS-R), to extract predictive relationships between spectral data and chemical properties [10]. For this, PLS-R analysis is a key tool in chemometrics, particularly for building predictive models in spectroscopic data calibration [11]. Its main advantage is the ability to handle datasets with many correlated variables, a common scenario in spectra obtained through NIR. As a multivariate regression method, PLS-R reduces the dimensionality of the original dataset by extracting latent variables (LVs), which are linear combinations of spectral variables that capture the most relevant information for prediction. Unlike other regression techniques, PLS-R not only maximizes the explained variance in the predictor variables but also optimizes their relationship with the response variable, enhancing the model’s predictive performance. To prevent overfitting and improve generalization, cross-validation is commonly applied, selecting the optimal number of components by minimizing prediction error. This approach is particularly useful when the number of predictors greatly exceeds the number of samples or when strong multicollinearity exists among independent variables.

Previous research has focused on the application of NIR combined with chemometrics to estimate total phenolic estimation or single antioxidant assays in intact olive drupes or olive oil [12,13]. However, olive leaves remain relatively unexplored in this regard, particularly evaluating three complementary methods (DPPH, ABTS, and ORAC). For this, this study is introduced as a comprehensive and innovative approach to rapidly assess the antioxidant capacity of *Olea europaea* L. leaves using near-infrared (NIR) spectroscopy combined with chemometric modeling. Thus, the aim of this study was to integrate multiple antioxidant assays, chemometric NIR modeling, and targeted HPLC phenolic profiling to explain the functional antioxidant potential of olive leaves, a valuable yet underexploited by-product of the olive oil industry.

## 2. Materials and Methods

### 2.1. Chemicals and Reagents

DPPH (2,2-Diphenyl-l-picrylhydrazyl), potassium persulfate (K_2_S_2_O_8_), ABTS (2,2-azino-bis [3-ethylbenzothiazoline-6-sulfonate]), fluorescein sodium salt, and AAPH (2,2′-azobis [2-methylpropionamidine] dihydrochloride) were purchased from Sigma-Aldrich (St Louis, MO, USA). Trolox (6-hydroxy-2,5,7,8-tetramethychroman-2-carboxylic acid) (purity > 97%) was purchased from Thermo Fisher Scientific (Waltham, MA, USA). The solvents and reagents employed were all HPLC purity or analytical grade. Ethanol and acetonitrile were purchased from Honeywell (München, Germany). Ultrahigh-purity water was produced using a Milli-Q system (Millipore, Bedford, MA, USA). The standards of oleuropein, hydroxytyrosol, verbascoside, and apigenin-7-glucoside were supplied from Sigma-Aldrich (Madrid, Spain).

### 2.2. Plant Material

During the 2022–2023 collection campaigns, a total of 120 samples of *Olea europaea* L. were collected from three Designations of Origin (DO) for extra virgin olive oil—Montes de Toledo, Campo de Calatrava, and Campo de Montiel—as well as from the “Marca Colectiva Gráfica de la Asociación Mesa del Aceite Sierra de Alcaraz.” All collection sites are located within areas included in the Castilla-La Mancha region, Spain. Leaves from each sample were collected from different trees and considered as independent biological replicates. The collected leaves were air-dried at room temperature (18 ± 3 °C) for three days. Subsequently, the samples were processed according to the method described by Martínez-Navarro et al. [4]. Briefly, dried leaves were ground using a knife mill (ARES FML-2000; Filtra Vibracion, Barcelona, Spain) and passed through a 0.5 mm mesh sieve until at least 95% of the total sample weight had passed through.

A moisture content analysis was conducted on the samples using a moisture balance equipped with a halogen lamp, specifically the XM-120 T model (Cobos, Barcelona, Spain) operating at a temperature of 105 °C. When moisture loss was less than 0.1% in 180 s, it was considered that the samples had reached constant mass at different locations.

### 2.3. Preparation of Extracts of Olea europaea L. Leaves

Leaf extracts were prepared following the method described by Martínez-Navarro et al. [4]. Briefly, 50 mg of olive leaf powder from each sample was mixed with 25 mL of distilled water and extracted using a domestic microwave oven (MS-2819W; Saivod, Madrid, Spain) at 800 W for 30 s. Then, the mixture was centrifuged at 3500 rpm for 10 min (Selecta, Barcelona, Spain), and the supernatant was filtered through a nylon syringe filter membrane with a pore size of 0.2 μm (Membrane Solutions, Plano, EEUU). The extracts from each sample were analyzed separately (n = 3).

### 2.4. Antioxidant Activity

#### 2.4.1. DPPH

Antioxidant capacity by DPPH method was measured according to the Brand-Williams method [14] with some modifications. Initially, 12 mg of DPPH was dissolved in 500 mL of ethanol. A measure of 290 µL of this solution was mixed with 10 µL of extract, previously diluted 1:2, into 96-well plates. Reaction mixture was incubated at 25 ± 0.2 °C and measured at 517 nm for 1 h, with readings every 5 min, in a Biotek Synergy H1 Multimode Reader (Palo Alto, CA, USA). Distilled water was used for the blank and Trolox was used as an antioxidant standard. The standard curve was constructed with five points between the interval 0.05 and 1 mM of Trolox. The analyses were performed in triplicate, and the DPPH results are expressed as mM Trolox equivalent.

#### 2.4.2. ABTS

The ABTS method was measured according to the Re method [15], with some modifications. Briefly, a solution of 38.4 mg of ABTS and 6.6 mg of potassium persulfate in 10 mL of distilled water was prepared 12–16 h before use and left at a cold temperature (4 °C) in the dark. To use it, a 1:100 dilution with ethanol was prepared. A measure of 290 µL of this solution was mixed with 10 µL of extract, previously diluted 1:2, into 96-well plates. Reaction mixture was incubated at 35 ± 0.2 °C and measured at 734 nm for 50 min, with readings every 5 min, in a Biotek Synergy H1 Multimode Reader (Palo Alto, CA, USA). Distilled water was used for the blank and Trolox was used as an antioxidant standard. The standard curve was constructed with five points between the interval 0.05 and 1 mM of Trolox. The analyses were performed in triplicate and the ABTS results are expressed as mM Trolox equivalent.

#### 2.4.3. ORAC

The ORAC method is based on fluorescence and was performed according to the Huang method [16] and Agilent protocol [17] with some modifications. Briefly, AAPH 295 mM was freshly prepared for each run in phosphate buffer (PBS, 75 mM). A 4 µM measure of fluorescein was prepared in PBS and then a dilution of 1:500 was prepared daily to use it. A measure of 150 µL of fluorescein was mixed with 25 µL of extract, previously diluted 1:50, in black-colored 96-well plates. After 5 min, 25 µL of AAPH was added using a multichannel pipette to start the reaction, following by shaking at maximum intensity for 10 s using a Biotek Synergy H1 Multimode Reader (Palo Alto, CA, USA). The equipment was programmed to record the fluorescence reading at an excitation wavelength of 485 nm and an emission wavelength of 528 nm, measuring from the top every 5 min using the autoscale option for gain optimization. The temperature was set at 37 ± 0.2 °C. Phosphate buffer was used for the blank and Trolox was used as the antioxidant standard. The standard curve was constructed with six points between the interval 6.25 and 100 µM Trolox. The analyses were performed in triplicate and the ORAC results are expressed as mM Trolox equivalent.

The antioxidant capacity for this method is normally calculated based on the area under the curve (AUC) (Equation (1)):

(1) $$AUC=\frac{F_{1}}{F_{1}}+\frac{F_{2}}{F_{1}}+\frac{F_{3}}{F_{1}}+\ldots \frac{F_{n}}{F_{1}}$$

where F_1_ is the fluorescence reading at the start of the reaction and F_2_ and F_3_ are second and third measurements, respectively, while F_n_ is the final measurement.

However, in this work, the AUC was adapted to consider only the start point and the end of the reaction (Equation (2)):

(2) $$AUC=\frac{F_{1}}{F_{n}}$$

where F_1_ is the fluorescence reading at the start of the reaction and F_n_ is the final measurement.

### 2.5. Near-Infrared Spectroscopy Measurements

Spectra of *Olea Europaea* L. leaves were obtained using Perkin Elmer Spectrum One FT-NIR equipment (Norwalk, CT, USA) coupled with a near-infrared reflectance accessory (NIR). The powdered olive leaf samples were deposited to completely cover the surface of a 1 cm^2^ quartz Petri dish (Perkin-Elmer, Norwalk, CT, USA). Flower samples were measured with a 16 cm^−1^ fixed resolution using a wavelength range of 750–2500 nm (13,333–4000 cm^−1^). All samples were scanned in duplicate.

### 2.6. Datasets Distribution

The distribution of the sets for calibration, validation, and prediction was carried out according to the Kennard–Stone (KS) algorithm [18]. This algorithm is based on the Euclidean distance between the variables of the X-axis, which corresponds to the obtained spectra. For the calibration, all samples were used (Set 1+2). Then, a 70% and 30% distribution, approximately, was performed for cross-validation (Set 1) and prediction (Set 2), respectively.

### 2.7. Spectral Preprocessing

Pretreatment combinations of the spectrum are conventionally used to improve the performance of the spectral model. To discover the best pretreatment for each antioxidant method, several spectral preprocessing algorithms were implemented using Spectrum-One software version 1.00 to perform data, including Savitzky–Golay smoothing (SGS), multiplicative scatter correction (MSC), first-order derivatives (1st D), second-order derivatives (2nd D), and standard normal variate (SNV). SGS is an averaging method that applies a polynomial fit to the data points. MSC is a transformation technique aimed at compensating for additive and/or multiplicative variations in spectral data. Derivative methods are used to address baseline distortions in the spectra, whereas SNV is a row-based transformation that centers and normalizes individual spectra [19]. The effectiveness of these preprocessing algorithms was assessed using Partial Least Squares Regression (PLS-R) analysis.

### 2.8. Model Evaluation

The model’s performance was examined using the determination coefficient (R^2^), root mean square error of cross-validation (RMSECV), and root mean square error of prediction (RMSEP), which was calculated when the model was tested on new data with known reference values. The residual predictive deviation (RPD) is defined as the ratio between the standard deviation of the reference dataset and either RMSEC (root mean square error of calibration), RMSECV, or RMSEP, calculated according to Wang et al.’s research [20] (Equations (3) and (4)). The table for the range of RPD values is Table S1.

(3) $$RMSE=\sqrt{\frac{\sum_{i=1}^{n}({f-y)}^{2}}{n}}$$

where “RMSE” is the root mean square error of calibration, cross-validation, or prediction, “f” is the prediction value, “y” is the measured value, and “n” is the number of samples.

(4) $$RPD=\frac{SD}{RMSE}$$

where “RMSE” is the root mean square error of calibration, cross-validation, or prediction and “SD” is the standard deviation of measured samples of calibration, cross-validation, or prediction.

### 2.9. Reversed-Phase High-Performance Liquid Chromatography with Diode Array Detection (RP-HPLC-DAD) Conditions

The chromatographic analysis was performed under conditions adapted from Martínez-Navarro et al. [4]. For each run, 20 μL of the filtered extract was injected into an Agilent 1200 Series HPLC system (Agilent, Palo Alto, CA, USA), fitted with a Diode Array Detector (model G1315D; Agilent) and operated using ChemStation software, version B.03.01 (Agilent). Separation was achieved on a reverse-phase C18 Brisa LC2 column (250 mm × 4.6 mm, 5 μm; Teknokroma, Barcelona, Spain), maintained at a constant temperature of 30 °C.

The mobile phase consisted of water (A) and acetonitrile (B). The gradient applied for solvent B was programmed as follows: 5% at 0 min; 20% at 10 min; 30% at 15–18 min; 50% at 36 min; 100% at 42–44 min; returning to 5% at 48–49 min. The flow was kept at 1 mL min^−1^ throughout the analysis. Compounds were detected with the DAD set at 280 nm. Identification relied on matching retention times and UV–Vis spectral data with those obtained from authentic reference standards (Sigma-Aldrich, Madrid, Spain). Quantification followed the external standard approach, using calibration curves established from five concentration levels of each reference compound (R^2^ = 0.99). Each sample was analyzed in triplicate, with one replicate from every extraction. Results are expressed as mg/g of leaf using percentage of moisture content previously measured.

## 3. Results

### 3.1. Antioxidant Potential of Olea europaea L. Leaves Across Sample Collection

Table 1 summarizes the antioxidant capacity, expressed as Trolox equivalent, of the *Olea europaea* L. samples assessed using three different assays: DPPH, ABTS, and ORAC.

For each method, the mean, standard deviation, and coefficient of variation (CV%) were calculated, with the CV (%) being the ratio of the standard deviation to the mean, multiplied by 100. Higher CV values in DPPH and ABTS than in ORAC may indicate that data were more concentrated in ORAC and only a few samples were at the lower and upper ends of the range data, while in DPPH and ABTS the data were more spread out. Related to the ranges, the DPPH values were between 0.42 and 0.96 mM, ABTS results spanned from 0.60 to 0.99 mM, and ORAC ranged between 0.94 and 4.10 mM. Martínez-Navarro et al. [20] studied antioxidant capacity in extracts of olive leaves from Greek and Spanish genotypes employing DPPH and ABTS assays, but very low results were obtained compared with this study. To evaluate predictive robustness, all samples (120) were used to build a calibration model and then split into two non-overlapping groups: 84 samples were reserved for cross-validation, while the remaining 36 were used for external prediction. This separation ensured that each sample contributed to only one phase of the model development.

### 3.2. Spectra Inspection

Before analyzing the spectral characteristics of *Olea europaea* L. leaves, it is essential to examine their phenolic composition. According to Martínez-Navarro’s research [21,22], the phenolic profile of olive leaves is composed of several groups of bioactive compounds. One of the primary groups is the secoiridoids, which are exclusive to the *Oleaceae* family. These compounds are structurally defined by the presence of an elenolic acid linked to glucosidic residue. Among them, oleuropein stands out as the predominant secoiridoid in olive leaves [23]. This compound consists of the characteristic secoiridoid structure conjugated with a hydroxytyrosol molecule. Another significant group is phenolic alcohols, to which hydroxytyrosol belongs. This compound can originate either from the enzymatic hydrolysis of oleuropein via lipase activity or from its aglycone derivatives [24], and it is recognized as a potent natural antioxidant [25]. Derivatives of hydroxycinnamic acids are also present in *Olea europaea* L. leaves, with verbascoside being the most representative compound. This molecule, found across various plant families, possesses two catechol groups that confer it a high antioxidant capacity [26]. Lastly, flavonoids constitute another relevant group within the phenolic profile of olive leaves. These compounds are characterized by two benzenic rings of six carbons linked by a heterociclical ring. Among flavonoids, flavones are the most prevalent subclass. Those quantified in *Olea europaea* L. leaves include apigenin-7-O-glucoside, luteolin-7-O-glucoside, and diosmetin-7-O-glucoside. Although their concentrations are relatively low in olive leaves, they have antioxidant capacity [25].

**Figure 1:**
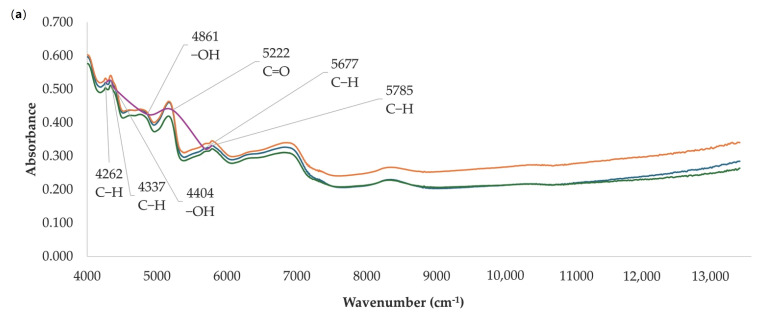

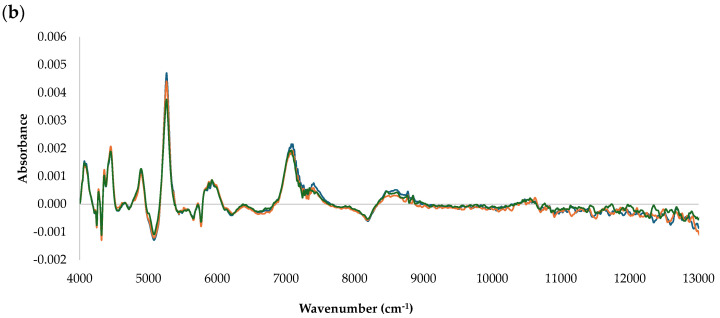
Near-infrared spectrum of some *Olea europaea* L. leaves analyzed (**a**) and their first derivative (**b**).

The spectra obtained from the *Olea europaea* L. leaf samples exhibit a similar tendency (Figure 1), with evident variations in the first derivative (Figure 1b) that could be attributed to the concentrations of chemical compounds previously mentioned, contributing to the broad variability observed in antioxidant responses. To conduct chemometric analysis on the NIR spectra, the absorption bands within the range of 13,333–4000 cm^−1^ were assessed to identify the functional groups responsible for the observed features. According to Can et al. [9], the spectra display a first overtone of C–H stretching at 5785 and 5677 cm^−1^, a second overtone of C=O at 5222 cm^−1^, an OH stretching and deformation band at 4861 cm^−1^, combination bands of NH and OH at 4404 cm^−1^, asymmetric and symmetric combination modes of CH stretching at 4337 cm^−1^, and CH_2_ bending vibrations at 4262 cm^−1^.

The bands at 5785 and 5677 cm^−1^, assigned to the first overtone of C–H stretching, are possibly linked to aliphatic and aromatic C–H groups present in the glucosidic moiety of secoiridoids such as oleuropein, as well as in the aromatic rings of flavonoids and hydroxytyrosol [9]. The signal at 5222 cm^−1^, corresponding to the second overtone of C=O stretching, could reflect the carbonyl groups characteristic of secoiridoids and their derivatives. The absorption at 4861 cm^−1^, related to OH stretching and deformation modes, can be attributed to the hydroxyl substituents in hydroxytyrosol, verbascoside, and flavone glycosides (apigenin-7-O-glucoside, luteolin-7-O-glucoside, diosmetin-7-O-glucoside) [27]. The band of OH at 4404 cm^−1^ may be associated with polyhydroxylated structures such as hydroxycinnamic acid derivatives and verbascoside, whose catechol groups contribute significantly to hydrogen-bonding interactions [26]. The bands at 4337 cm^−1^ (C–H stretching combination modes) and 4262 cm^−1^ (CH_2_ bending vibrations) correspond to methylene and aromatic C–H groups present in the flavonoid backbone and in the elenolic acid residue of secoiridoids [28].

### 3.3. NIR Calibration and Validation of Antioxidant Capacity

For the three antioxidant capacity methods (DPPH, ABTS and ORAC), PLS-R was used to perform the calibration model with the more appropriate pretreatment to increase the performance of the predictive models in the selected spectral range, since the original spectral model achieved a mean R_c_ and R_cv_ of 0.07 (DPPH), 0.04 (ABTS), and 0.03 (ORAC). The same pretreatment was applied for the three methods across the calibration, cross-validation, and prediction processes, and the four best pretreatments are summarized in Table 2**.** For all the models, the entire infrared spectral range (13,333–4000 cm^−1^) was selected after eliminating the redundant spectra. This was mainly because to the high variety of compounds present in *Olea europaea* L. leaves [29], making it difficult to define a more limited spectral range for model development due to the complex mix of functional groups and bond types present in them.

For DPPH, in ascending order of performance, a 1st derivative (1st D) with 19 points was applied, followed by Savitzky–Golay smoothing (SGS) (5 points) and multiplicative scatter correction (MSC), to correct light scattering effects. This resulted in an R^2^_cv_ of 0.80, an R^2^_p_ of 0.82, and an RMSEP of 0.087. Applying 2nd D (13 points), SGS (9 points), and SNV yielded an R^2^_cv_ of 0.85, R^2^_p_ of 0.86, and RMSEP of 0.078. To further reduce noise, higher derivatives and additional SGS points were tested. A 2nd derivative (2nd D) with 19 points, followed by SGS (5 points) and SNV, resulted in an R^2^_cv_ of 0.92 and R^2^_p_ of 0.90, and an RMSEP of 0.073. To assess the impact of different normalization techniques, the same preprocessing was tested using standard normal variate (SNV) instead of MSC, and despite the differences in normalization methods to correct light scatterings, the results remained identical (Table 2). Compared to the first two pretreatments, this approach yielded improved RMSEP values, demonstrating the benefits of using a higher derivative and more SGS points, making it the optimal pretreatment for DPPH analysis. Higher derivatives and additional SGS points were tested, leading to poorer results due to excessive smoothing or spectral distortion. The RMSEC and RMSECV values were found to be quite similar between different pretreatments.

The RPD values obtained for calibration were excellent due to an R^2^ value of 1. The validation values ranged from 1.89 to 4.1 and prediction values ranged from 2.02 to 3.01 (Table 2). During calibration, for all pretreatments, extraordinary results were achieved. In cross-validation, the highest RPD value was obtained when applying a 2nd D with 19 points, followed by SGS with 5 points and SNV or MSC. For prediction, the highest RPD was achieved when a 2nd D with 19 points was considered, followed by SGS with 5 points.

For all the DPPH pretreatments (Table 2), the RPD values observed for prediction ranged from 2.02 to 3.01, with the best pretreatment surpassing the threshold of 3.0, which is generally accepted as suitable for quality control purposes according to Table S1 [30]. Lower RPD values obtained with other pretreatments indicate their limited applicability for quantitative purposes, although they may still be useful for screening. According to Moncada et al. [31], the DPPH assay showed the best quality parameters compared to the other antioxidant assays. Thus, the best performing model for DPPH estimation was achieved using a 2nd derivative with 19 smoothing points, followed by Savitzky–Golay smoothing (5 points) and SNV normalization. This combination yielded a cross-validation coefficient (R^2^_cv_) of 0.92, a prediction coefficient (R^2^_p_) of 0.90, and an RMSEP of 0.073. These values indicate high model robustness and accuracy. Wu et al. [19] studied DPPH in bamboo leaf extracts, who reported R^2^ values of between 0.80 and 0.88 for bamboo leaf extracts using NIR and PLS, supporting the potential of this technique for predicting antioxidant related parameters in this matrix. In contrast, other pretreatments (such as 1st derivative with MSC or lower SGS points) showed slightly inferior predictive performance, with RMSEP values ranging from 0.078 to 0.087 and R^2^_p_ values from 0.82 to 0.86. The progressive improvement in model performance with higher order derivatives and optimized smoothing suggests better resolution of overlapped signals, a common challenge in NIR analysis of complex matrix [32]. Notably, both SNV and MSC normalization yielded similar results, aligning with previous findings that both techniques effectively correct for multiplicative light scattering effects [33].

For the ABTS method, the same pretreatments applied in the DPPH method were used to identify differences or similarities between them. These pretreatments, which were summarized in Table 2, were consistently applied across calibration, cross-validation, and prediction processes. In order of increasing performance, a 1st derivative (1st D) with 19 points was first applied, followed by SGS (5 points) and MSC to correct light scattering effects. This resulted in an R^2^_cv_ of 0.79, an R^2^_p_ of 0.81, and an RMSEP of 0.091. Compared to the DPPH method, the ABTS one yielded lower values (Table 2). Since higher derivative and SGS points can smooth the spectrum over, lower values were tested. Using a 2nd D (13 points), SGS (9 points), and SNV resulted in an R^2^_cv_ of 0.81, R^2^_p_ of 0.82, and an RMSEP of 0.087. Although a higher R^2^_p_ was obtained compared to the first pretreatment, RMSEC and RMSEP values also decreased. Overall, the ABTS method produced poorer results compared to DPPH when the same pretreatment was applied (Table 2).

Given these findings, a 2nd D (5 points), followed by SGS (19 points) and SNV, was applied, yielding an R^2^_cv_ of 0.85, R^2^_p_ of 0.88, and RMSEP of 0.064. Based on these results, this pretreatment was considered optimal for ABTS analysis. As in the DPPH method, higher derivative and additional SGS points were tested, but they resulted in poorer outcomes due to excessive smoothing or spectral distortion. The RMSE values (RMSEC, RMSECV, and RMSEP) remained relatively similar across different pretreatments (Table 2). Regarding RPD values, the range for calibration was excellent due to an R^2^ value of 1. For cross-validation, the RPD values ranged between 1.79 and 3.14, and for prediction ranged between 1.92 and 2.90. The best cross-validation value was achieved using a second derivative with 5 points, followed by SGS with 19 points and SNV or MSC. The same pretreatment also yielded the best prediction RPD value. Thus, based on the established criteria for selecting the best model, the optimal pretreatment for ABTS analysis was identified as a 2nd derivative (5 points), SGS (19 points), and either SNV or MSC, as it yielded the highest R^2^, RPD_p_, and RMSE values (Table 2). Thus, according to Table S1 and observing the RPD_p_ values obtained for each pretreatment in Table 2, it could be indicated that the pretreatments were generally poor, being only quite effective and suitable for quality control of the previously mentioned optimal pretreatment.

Applying the same preprocessing steps as in the DPPH model (1st derivative, SGS and MSC), the ABTS model initially produced a moderate R^2^_p_ of 0.81 and RMSEP of 0.091. Despite similarities in data treatment, the ABTS model consistently underperformed compared to the DPPH model. This finding aligns with previous observations that ABTS, though widely used, may be less specific in reacting with certain antioxidant compounds, especially those predominant in olive leaves, and may therefore yield more variable spectroscopic responses [34]. Subsequent improvement using a 2nd derivative (13 points) and SNV provided slight gains (R^2^_p_ = 0.82, RMSEP = 0.087), but it was not until the pretreatment combining a 2nd derivative (5 points), SGS (19 points), and SNV was applied that the best performance was achieved (R^2^_p_ = 0.88, RMSEP = 0.064). This result confirms that lower derivative orders with increased smoothing points can better capture subtle spectral differences while minimizing noise, a strategy also supported by Rinnan et al. [32] in spectroscopic modeling of biological samples.

Interestingly, despite a similar preprocessing pipeline, ABTS modeling yielded slightly lower RPD values than DPPH. RPD values for prediction ranged from 1.92 to 2.90, with only the best pretreatment reaching a value close to the threshold considered acceptable for quality control applications (RPD_p_ ≈ 3.0). This trend further supports the notion that ABTS may be less sensitive in detecting NIR relevant antioxidant spectral signals, possibly due to differences in radical reactivity mechanisms compared to DPPH, which tends to correlate more strongly with phenolic content [8]. Therefore, while the ABTS assay can be modeled using NIR spectroscopy with acceptable precision, its predictive capacity is more limited than DPPH under the same chemometric strategies. The optimal model for ABTS (2nd D—5 points, SGS—19 points, SNV) should be considered suitable for semiquantitative applications or screening purposes, but additional improvements or complementary methods (e.g., integrating UV-visible or MIR spectroscopy) might be required to reach higher robustness for routine quality control purposes.

Finally, for the ORAC method, the same procedure was followed as for the DPPH and ABTS methods. Thus, the same pretreatments applied in the DPPH and ABTS method were applied to identify differences or similarities between them. Results of these pretreatments in ORAC yielded low values, where only one of them gave good results (Table 2). In fact, different pretreatments for calibration, cross-validation, and prediction processes were tested.

In ascending order, a 1st derivative (1st D) with 19 points was applied, followed by SGS (5 points) and MSC to correct light scattering effects. This resulted in an R^2^_cv_ of 0.50, an R^2^_p_ of 0.52, and an RMSEP of 0.98. Compared to the DPPH and ABTS methods, the ORAC one yielded poorer results. Along with the above, a 2nd D with 13 points and SGS with 9 points and SNV was applied, resulting in an R^2^_cv_ of 0.70, an R^2^_p_ of 0.73, and an RMSEP of 0.55. Compared to the DPPH and ABTS methods, the values were very low, but the performance increased with a 2nd D. Finally, a 2nd D with 19 points, SGS of 5 points, and SNV was applied, yielding results of an R^2^_cv_ of 0.88, an R^2^_p_ of 0.87, and an RMSEP of 0.47. The same pretreatment was applied using MSC instead of SNV, yielding identical results. For the RPD values, due to an R^2^ value of 1, the calibration was excellent across pretreatments. For cross-validation, values ranged between 0.82–3.50 and 0.90–2.69 for prediction. According to the RPD_p_ values (Table 2) and the information summarized in Table S1, the pretreatments for this method would fall below 2.0, indicating that its application would not be recommended. However, the last pretreatment could be applied to screening.

The difference among the DPPH and ABTS methods versus ORAC could be attributed to the fact that ORAC is based on fluorescence, which tends to produce more variable and less consistent results due to its high sensitivity to abiotic factors (temperature, concentration, light, etc.), while DPPH and ABTS are based on absorbance, making them more stable and reliable and, therefore, less sensible to abiotic factors [35].

Predictive modeling of antioxidant capacity using the ORAC assay in *Olea europaea* L. leaf extracts, in conjunction with NIR spectroscopy and chemometric pretreatments, exhibited lower accuracy compared to DPPH and ABTS models. Following the same preprocessing pipeline applied in the previous assays, three spectral pretreatment strategies were evaluated (Table 2). Among these, only one resulted in acceptable predictive statistics. Specifically, the best performing model applied a 2nd derivative (19 points), Savitzky–Golay smoothing (5 points), and SNV normalization, yielding an R^2^_cv_ of 0.88, R^2^_p_ of 0.87, and RMSEP of 0.47. Nevertheless, the corresponding RPD_p_ value (2.69) remained below the critical threshold of 3.0 required for robust quantitative application, suggesting that this model may only be suitable for screening purposes. All other models yielded substantially lower performance (R^2^_p_ < 0.73), consistent with the typically lower reproducibility of ORAC-based predictions [35,36]. The limited predictive capacity of the ORAC assay in this context can be partially attributed to the methodological nature of the test. ORAC relies on the fluorescence decay of fluorescein in the presence of peroxyl radicals generated by AAPH. Unlike the DPPH and ABTS methods, which are based on direct absorbance and relatively stable radical systems, the ORAC assay involves a time resolved kinetic process that is more sensitive to environmental conditions such as temperature and pH, which affect the decomposition rate of AAPH and the stability of fluorescein [36]. Fluorescence variability is influenced by external light sources or detector sensitivity. Fluorescein photodegradation can artificially inflate or deflate antioxidant capacity readings, especially in low light-controlled settings [37]. Moreover, olive leaf extracts possess a complex matrix of phenolic compounds (e.g., oleuropein, hydroxytyrosol, verbascoside), which may interact with fluorescein or interfere with peroxyl radical quenching differently than in absorbance-based assays [38].

In general, the prediction of antioxidant capacity in *Olea europaea* L. leaf extracts using NIR spectroscopy showed promising but assay-dependent performance. When benchmarked against the three methods, raw spectral models exhibited poor correlation (R_c_ = 0.07), highlighting the necessity of advanced pretreatments to reduce baseline shifts, light scattering, and overlapping signals. After applying optimized preprocessing, DPPH predictive accuracy improved considerably, confirming the ability of NIR to capture the complexity of olive leaves, which contain a wide range of phenolic compounds such as oleuropein, hydroxytyrosol, verbascoside, apigenin, and other flavonoids [38]. Comparable models built with the ABTS assay achieved slightly lower predictive performance, though they reinforced the need to retain the full spectral range (13,333–4000 cm^−1^) to account for the overlapping features arising from the structurally diverse phenolic matrix. In contrast, ORAC-based predictions were less robust overall. Of the three preprocessing strategies tested (Table 2), only the combination of second derivative (19 points), Savitzky–Golay smoothing (5 points), and SNV normalization yielded acceptable statistics (R^2^_cv_ = 0.88, R^2^_p_ = 0.87, RMSEP = 0.47). Yet, the corresponding RPD_._ value (2.69) did not surpass the 3.0 threshold considered necessary for reliable quantification, indicating that ORAC-derived models may be limited to screening applications.

The findings of this study are largely consistent with previous research on the application of near-infrared spectroscopy for the rapid assessment of antioxidant capacity in plant matrices, while offering several important advancements in both experimental scope and mechanistic understanding. First, Can et al. [9] demonstrated the potential of NIR and mid-infrared spectroscopy (MIR) to quantify oleuropein, total phenolics, and flavonoids in olive leaves, achieving strong correlations (R^2^ ≈ 0.85). However, their work was limited to the determination of individual chemical constituents. In contrast, the present study extends this approach by directly predicting functional antioxidant activity through three reference assays (DPPH, ABTS, and ORAC), providing a methodological advance by linking spectral features to integrated biological responses rather than isolated chemical parameters. Similarly, Vallinger et al. [39] used NIR coupled with PLS-R to estimate bioactive compounds activity using Folin–Ciocalteu and DPPH in olive leaf extracts, reporting R^2^ values between 0.90 and 0.96 but not RPD values. The DPPH models obtained in the present research achieved comparable predictive performance (R^2^_p_ = 0.90; RPD = 3.01), confirming the robustness of NIR chemometric modeling in phenolic-rich plant materials and supporting the hypothesis that O–H and C–H overtone absorptions from aromatic structures dominate the NIR signal associated with antioxidant potential.

In line with research by Pompeu et al. [40] and Li et al. [41], the present work also shows that models based on the ORAC assay exhibit lower predictive capacity due to the high sensitivity of fluorescence measurements to temperature, pH, and photodegradation of fluorescein. Although acceptable calibration results were obtained (R^2^_cv_ ≈ 0.88), the RPD_p_ value lower than 3 indicates limited quantitative reliability, confirming that ORAC-based NIR models are suitable mainly for screening applications. Conversely, absorbance-based methods such as DPPH and ABTS yielded slightly more stable and reproducible NIR models, according to the RPD_p_ obtained. This highlights that the choice of the reference assay critically affects NIR predictive performance, as each method involves distinct reaction mechanisms (electron transfer, hydrogen atom transfer, mix of two, peroxyl radicals, etc.). Furthermore, Bellincontro et al. [28] reported the utility of NIR for estimating phenolic compounds during the ripening of olives for oil production but did not evaluate antioxidant activity directly. The present results complement their findings by demonstrating that the same spectral information can accurately predict antioxidant response, confirming the potential of NIR as a functional analytical tool rather than merely compositional. Finally, Martínez-Navarro et al. [21] evaluated olive leaves from Greek and Spanish genotypes using DPPH and ABTS assays but without NIR analysis. The antioxidant capacities reported (0.42–0.96 mM Trolox for DPPH; 0.60–0.99 mM for ABTS) were higher, likely due to differences in regional origin, because the extraction technique (microwave-assisted aqueous extraction) was the same. This comparison suggests that expanding the calibration to include samples from multiple varieties and harvest seasons would further improve model generalization. Overall, this study represents the first comprehensive NIR-based comparison of three antioxidant assays (DPPH, ABTS, and ORAC) in olive leaves, integrating with HPLC-DAD phenolic profiling. This multidimensional approach provides a deeper understanding of how the chemical composition and optical properties of the matrix determine antioxidant behavior, paving the way for robust and transferable NIR models for sustainable quality control and valorization of olive by-products.

Although this work demonstrates the potential of near-infrared spectroscopy combined with chemometric analysis to predict the antioxidant capacity of *Olea europaea* L. leaves, certain limitations should be acknowledged. First, the dataset analyzed originated from a Castilla-La Mancha Spanish region, which could restrict the applicability of the developed models to other regions, cultivars, or growing conditions. From an instrumental standpoint, the observed dependence on specific spectral preprocessing combinations (derivatives, smoothing, and normalization) suggests that the models may require recalibration when applied to other instruments or optical configurations. Moreover, the use of the full spectral range (13,333–4000 cm^−1^) may have introduced redundancy and noise, contributing to potential overfitting during calibration (R^2^ = 1). Regarding the reference assays, the intrinsic methodological differences among DPPH, ABTS, and ORAC influenced the predictive performance of the models. In particular, the ORAC-based model showed limited robustness (RPD < 3), likely due to the high sensitivity of fluorescence-based measurements to experimental factors such as temperature and pH, restricting its use to screening applications. Finally, the absence of an independent external validation using samples from new harvesting campaigns or different storage conditions prevents full confirmation of the model’s generalizability.

### 3.4. NIR Prediction Models for Antioxidant Capacity

**Figure 2:**
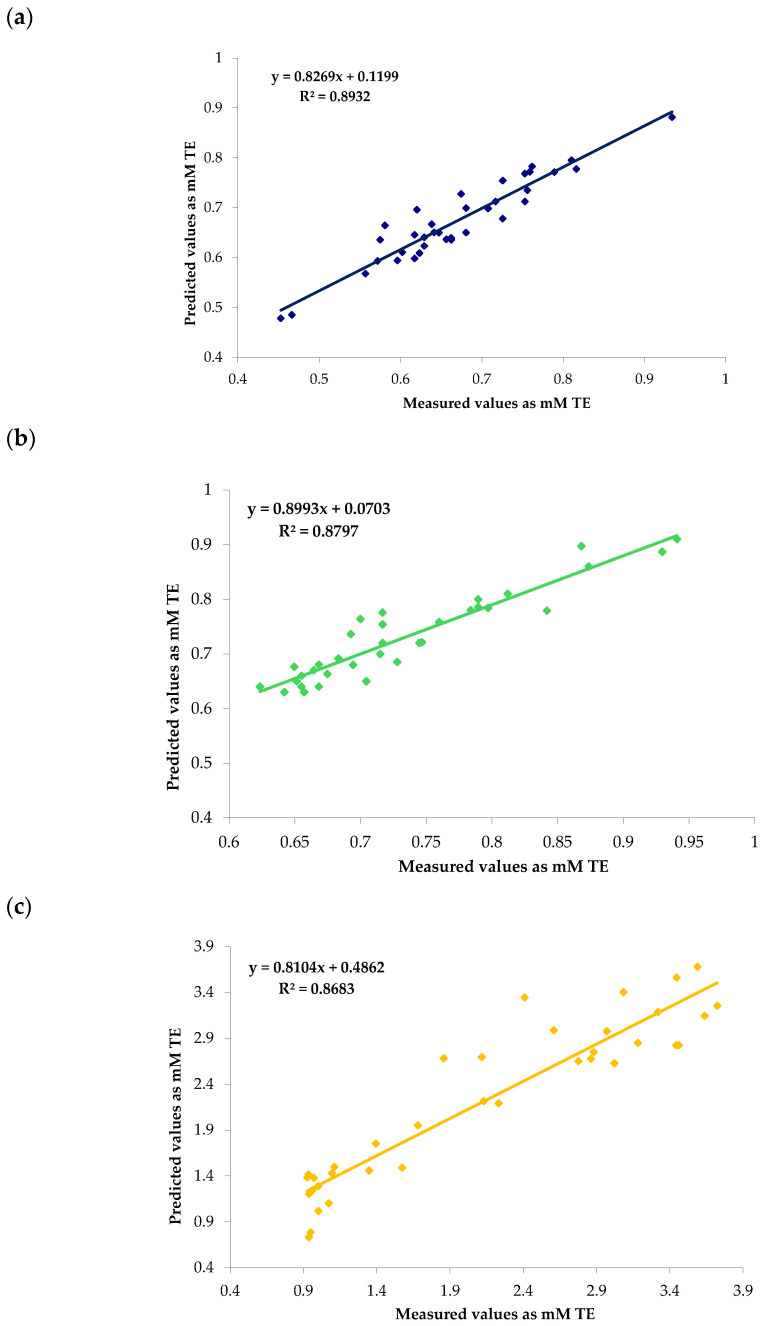
Scatter plot obtained with antioxidants results versus values predicted by the NIR in *Olea europaea* L. leaves using PLS-R: (**a**) DPPH; (**b**) ABTS; (**c**) ORAC.

Figure 2 shows scatter plots of the values of olive leaf antioxidant capacity by NIR versus the values obtained with DPPH, ABTS, and ORAC assays using the best calibration model (Table 2). The three presented models demonstrate positive linear correlations. Nonetheless, distinct differences in accuracy and robustness were observed among the models, allowing for a critical evaluation of their practical applicability and statistical performance.

The DPPH model (Figure 2a) exhibited the highest coefficient of determination (R^2^ = 0.8932), indicative of a strong predictive capability for antioxidant activity using this method. The slope (0.8269) approximates unity, and the minimal dispersion of data points around the regression line supports the high robustness, reliability, and stability of the model against small experimental variations. Although the intercept deviates slightly from zero (0.1199), this bias is marginal and acceptable within analytical contexts. The ABTS model (Figure 2b) displayed a comparably good fit (R^2^ = 0.8797), with a slope (0.8993) closer to unity than that of DPPH, suggesting a proportionally accurate prediction to measurement relationship. However, increased scatter around the regression line and a narrower calibration range may limit its robustness, especially in samples exhibiting significant chemical or structural heterogeneity, such as complex plant extracts from olive leaves. Its robustness could be improved by adjusting the calibration or expanding the data range. Finally, the ORAC model (Figure 2c) yielded a similar R^2^ value (0.8683). However, a pronounced dispersion of residuals was observed, particularly at elevated antioxidant capacity values that could be slightly underestimated. This variability could be attributable to the intrinsic characteristics of the ORAC assay, which relies on fluorescence detection, a technique sensitive to fluctuations in experimental parameters including temperature, pH, and light exposure. Moreover, the elevated intercept (0.4862) suggests more substantial systematic bias. Less robustness compared with two other models suggests that it could be a reliable model for prediction and screening applications, with the potential for improvement.

Considering the results obtained, while the ORAC-based model may be employed for preliminary screening purposes, its utility for precise quantitative prediction is limited by the absence of rigorous standardization of assay conditions. On the other hand, the DPPH model outperformed the others in predictive accuracy and robustness, closely followed by ABTS. The comparatively inferior performance of the ORAC model highlights the impact of the chosen antioxidant assay on the quality and reliability of NIR calibration models. Notably, absorbance-based methods (DPPH and ABTS) confer greater stability and reproducibility relative to fluorescence-based approaches such as ORAC.

### 3.5. Correlation Between Phenolic Compounds and Antioxidant Capacity in NIR Samples

As has been shown, the calibration of the different antioxidant capacity methods was different, mainly due to the intrinsic properties of each reagent. The compounds present in the olive leaf samples, although phenolic, show structural differences that may affect the reaction mechanisms. To demonstrate the possible relationships between each analysis method and the main phenolic compounds, a correlation analysis (Table 3) was performed with the antioxidant capacity determined through the three complementary methods used in this study (DPPH, ABTS, and ORAC), each based on distinct radical species and reaction mechanisms [8] and the phenolic composition previously determined by HPLC-DAD by Escobar-Talavera et al. [42]. This integrated approach would enable the estimation of the specific contribution of each compound to the overall antioxidant activity of the extracts.

Oleuropein showed significant positive correlations with both DPPH (r = 0.383; *p* < 0.01) and ORAC (r = 0.524; *p* < 0.01), but not with ABTS (Table 3). This suggests that its antioxidant activity is more effectively captured by assays based on hydrogen atom transfer, particularly ORAC, which mimics biological radical conditions. Its secoiridoid structure and multiple hydroxyl groups likely enhance its reactivity toward peroxyl and DPPH radicals. Previous work by Platzer et al. showed results that DPPH assay appeared to depend mainly on the number of OH groups [43]. The lack of correlation with ABTS may be due to the assay’s reliance on electron transfer, a mechanism where oleuropein is possibly less efficient, due to steric or solubility limitations.

Hydroxytyrosol showed the same behavior as oleuropein: it showed significant positive correlations with DPPH (r = 0.365; *p* < 0.01) and ORAC (r = 0.411; *p* < 0.01), but no correlation with ABTS (Table 3). This result is consistent with its chemical structure, which includes two hydroxyl groups that favor hydrogen atom transfer (HAT), a mechanism predominant in ORAC and partially in DPPH [44]. Hydroxytyrosol is also a remarkable molecule, ranking as the second most potent natural antioxidant after gallic acid [45]. The lack of correlation with ABTS may be due to steric hindrance or solvent effects, as the ABTS radical cation is more sensitive to solubility and polarity factors, and hydroxytyrosol may not react as efficiently in that assay environment. In contrast, hydroxytyrosol hexoside showed weak and non-significant correlations with DPPH and ORAC, and only modest correlation with ABTS (r = 0.207; *p* < 0.05) (Table 3). The presence of sugar in this molecule can hinder radical scavenging activity by decreasing the accessibility of the active phenolic hydroxyls or modifying the compound’s reactivity through steric or solvation effects [46], explaining its lower performance.

Verbascoside displayed a weak and non-significant correlation with DPPH and ORAC but a significant positive correlation with ABTS (r = 0.244; *p* < 0.01) (Table 3). This suggests that verbascoside is more reactive toward the ABTS^•+^ radical cation, which is more stable in aqueous environments and can interact with polar glycosylated polyphenols. Verbascoside also contains caffeic acid, which can contribute to its moderate ABTS reactivity, although the overall structure may limit its reactivity toward neutral soluble radicals like DPPH or peroxyl radicals in ORAC. Platzer et al. also studied phenolic substances and differences between DPPH and ABTS, achieving higher values in ABTS assay for phenolic acids like hydroxycinnamic acids [43].

Interestingly, apigenin-7-glucoside was not significantly correlated with DPPH or ORAC and showed a weak negative correlation with ABTS (r = −0.184; *p* < 0.05). As a glycosylated flavone, apigenin-7-glucoside is known for having low antioxidant activity in chemical assays, particularly in aqueous systems, due to poor solubility in some radical media [47]. Its poor reactivity with ABTS^•+^ may indicate a lower concentration and minimal antioxidant effect, which could explain the observed negative trend.

Overall, oleuropein and hydroxytyrosol, due to their concentration, remain the main contributors to antioxidant capacity in the NIR samples, according to Romero-Márquez et al. [48], particularly in the DPPH and ORAC methods, which rely more heavily on hydrogen atom transfer (HAT) and peroxyl radical scavenging mechanisms.

## 4. Conclusions

This research demonstrates the feasibility of near-infrared spectroscopy combined with chemometric techniques as a rapid, non-destructive, and environmentally friendly tool for predicting the antioxidant capacity of *Olea europaea* L. leaves. Among the evaluated assays, the DPPH model showed the highest accuracy and robustness, followed by ABTS, whereas the ORAC model exhibited limited performance and appeared to be suitable only for screening purposes. These findings highlight that the choice of reference assay strongly influences the quality of the developed NIR models. The observed correlations between major phenolic compounds, such as oleuropein and hydroxytyrosol, and antioxidant capacity values confirm the predominant role of these metabolites in the overall activity of the extracts. The structural diversity of phenolic compounds and the nature of the radical species used in each assay justify the differences in predictive performance, underscoring the importance of validating models with multiple methods for a more comprehensive characterization.

This study compared different antioxidant assays to assess how their underlying chemical mechanisms affect the predictive performance of NIR models. Correlations between antioxidant capacity and specific phenolic compounds revealed key metabolites driving antioxidant behavior. The NIR models demonstrated strong potential as rapid, non-destructive, and eco-efficient tools for evaluating olive leaves, though limited sample diversity and lack of external validation constrain applicability. Expanding and validating these models could enable sustainable valorization of olive byproducts.

## Figures and Tables

**Table 1:** Statistical data of antioxidant activity (mM Trolox) of extracts of *Olea europaea* L. leaves.

Antioxidant Method	Data Sets	N	Range	Mean ± SD	CV (%)
DPPH	Set 1+2	120	0.42–0.96	0.69 ± 0.23	33.32
	Set 1	84	0.42–0.96	0.69 ± 0.22	32.74
	Set 2	36	0.45–0.94	0.70 ± 0.20	31.33
ABTS	Set 1+2	120	0.60–0.99	0.74 ± 0.26	30.67
	Set 1	84	0.60–0.99	0.74 ± 0.25	30.43
	Set 2	36	0.62–0.96	0.76 ± 0.23	29.14
ORAC	Set 1+2	120	0.94–4.10	2.72 ± 0.55	12.86
	Set 1	84	0.94–4.10	2.77 ± 0.56	12.12
	Set 2	36	0.94–3.70	2.68 ± 0.52	12.56

N = number of samples used in each set; SD = standard deviation; CV: coefficient of variation calculated as SD/Mean.

**Table 2:** NIR statistical parameters for the calibration, cross-validation, and prediction data of the measured extracts of *Olea europaea* L. leaves.

Antioxidant Method	Spectral Range (cm^−1^)	Pre-Process	Data Sets	R^2^	RMSEC	RMSECV	RMSEP	RPD
DPPH	13,333–4000	MSC- 1.19.5	Set 1+2	1.00	0.000			
			Set 1	0.80		0.090		1.89
			Set 2	0.82			0.087	2.02
		SNV- 2.13.9	Set 1+2	1.00	0.000			
			Set 1	0.85		0.081		2.01
			Set 2	0.86			0.078	2.30
		MSC- 2.19.5	Set 1+2	1.00	0.000			
			Set 1	0.92		0.067		4.10
			Set 2	0.90			0.073	3.01
		SNV- 2.19.5	Set 1+2	1.00	0.000			
			Set 1	0.92		0.067		4.10
			Set 2	0.90			0.073	3.01
ABTS	13,333–4000	MSC- 1.19.5	Set 1+2	1.00	0.000			
			Set 1	0.79		0.094		1.79
			Set 2	0.81			0.091	1.92
		SNV- 2.13.9	Set 1+2	1.00	0.000			
			Set 1	0.81		0.090		1.85
			Set 2	0.82			0.087	2.13
		SNV- 2.5.19	Set 1+2	1.00	0.000			
			Set 1	0.85		0.073		3.14
			Set 2	0.88			0.064	2.90
		MSC- 2.5.19	Set 1+2	1.00	0.000			
			Set 1	0.85		0.073		3.14
			Set 2	0.88			0.064	2.90
ORAC	13,333–4000	MSC- 1.19.5	Set 1+2	1.00	0.000			
			Set 1	0.50		0.98		0.82
			Set 2	0.52			0.99	0.90
		SNV- 2.13.9	Set 1+2	1.00	0.000			
			Set 1	0.70		0.62		1.42
			Set 2	0.73			0.55	1.63
		SNV- 2.19.5	Set 1+2	1.00	0.000			
			Set 1	0.88		0.36		3.50
			Set 2	0.87			0.47	2.69
		MSC- 2.19.5	Set 1+2	1.00	0.000			
			Set 1	0.88		0.36		3.50
			Set 2	0.87			0.47	2.69

MSC (Multiplicative Scatter Correction) and SNV (Standard Normal Variate): preprocessing applied to correct for light scattering in the samples. The three-digit code (a.b.c.) where ‘a’ refers to the number of the derivative (1 and 2), ‘b’ is the interval over which the derivative is calculated (5, 9, and 13), and ‘c’ corresponds to the number of data points in a smoothing (5, 13, and 9). R^2^: coefficient of determination; RMSEC: root mean squared error of calibration; RMSECV: root mean squared error of cross validation; RMSEP: root mean squared error of prediction; RPD: calculated according to Wang et al. (SD/RMSE).

**Table 3:** Pearson correlation coefficients (r) between principal phenolic compounds found in *Olea europaea* L. leaves and antioxidant assays.

	DPPH	ABTS	ORAC
Oleuropein	0.383 **	0.011	0.524 **
Hydroxytyrosol	0.365 **	−0.050	0.411 **
Hydroxytyrosol hexoside	0.125	0.207 *	−0.052
Verbascoside	0.007	0.244 **	−0.165
Apigenin-7-glucoside	0.042	−0.184 *	−0.095

Significant correlation values are printed in bold according to Fisher’s LSD test (* *p* value < 0.05; ** *p* value < 0.01).

## Data Availability

The data presented in this study are available upon request from the authors due to privacy.

## Supplementary Materials

The following supporting information can be downloaded at https://www.mdpi.com/article/10.3390/antiox14101246/s1: Table S1: Degrees of merit for the RPD that are appropriate to the application of NIR spectroscopy.
