# Supplementary material for: Potential Rapid Quantification of Antioxidant Capacity of Olea europaea L. Leaves by Near-Infrared Spectroscopy Using Different Assays
Source: Antioxidants (Basel). 2025 Oct 17;14(10):1246. doi: 10.3390/antiox14101246 (PMC12561891; doi:10.3390/antiox14101246)
Supplement: Supplementary file 1 [file antioxidants-14-01246-s001.zip › antioxidants-3932772-supplementary.pdf]

Suplementary Material

Table S1. Degrees of merit for the RPD that are appropriate to the application of NIR spectroscopy.

RPD value	Classification	Application
**0.0-1.9**	Very poor	Not recommended
**2.0-2.4**	Poor	Rough screening
**2.5-2.9**	Fair	Screening
**3.0-3.4**	Good	Quality control
**3.5-4.0**	Very good	Process control
**>4.1**	Excellent	Any application
